# Ecotoxicology of Mercerized Cellulose–Treated Tannery Effluents Using *Salvinia auriculata* (Salviniaceae) as a Bioindicator

**DOI:** 10.1002/tox.24550

**Published:** 2025-06-19

**Authors:** Alex Rodrigues Gomes, Letícia Paiva de Matos, Abner Marcelino Silva, Abraão Tiago Batista Guimrães, Thiarlen Marinho da Luz, Rafaela Ribeiro de Brito, Aline Sueli Lima de Rodrigues, Juraci Alves de Oliveira, Ivandilson Pessoa Pinto de Menezes, Guilherme Malafaia

**Affiliations:** ^1^ Laboratory of Toxicology Applied to the Environment Goiano Federal Institute – Urutaí Campus Urutaí Brazil; ^2^ Universidad de Alcalá (UAH) Alcalá Spain; ^3^ Federal University of Viçosa Viçosa Brazil; ^4^ Federal Institute of Ceará, IFCE, Acopiara Cumpus Acopiara Brazil

**Keywords:** biochemical biomarkers, chromium, ecotoxicology, effluent treatment, *Salvinia auriculata*, tannery effluents

## Abstract

Given the growing concern over the environmental impacts of industrial effluents, particularly from tanneries, assessing the ecotoxicological risks associated with these effluents, even after remediation treatments, is crucial. Therefore, we aimed to evaluate the potential effects of exposure to raw and treated tannery effluents with mercerized microcrystalline cellulose particles (MCPs) on *Salvinia auriculata* . This study addresses the need for sustainable treatment alternatives that can reduce toxicity while assessing the residual impacts on aquatic plants. Plants were exposed to effluent dilutions (0.3% and 3.1%) for 15 days under controlled conditions. Biomarkers related to growth, photosynthesis (chlorophyll *a*, *b*, and total), oxidative stress (ROS, MDA, nitrite), and antioxidant enzyme activities (SOD, CAT, and SOD/CAT ratio) were analyzed. Although MCPs reduced chromium concentrations, treated effluents still caused significant toxicity, with root growth inhibition reaching 40% and chlorophyll *a* decreasing by over 30%. Principal Component Analysis (PCA) and Cluster Analysis revealed clear group separation, driven by Cr accumulation and changes in key physiological and biochemical markers. These findings highlight the partial effectiveness of MCPs and the importance of including ecotoxicological endpoints when evaluating treatment technologies. Although MCPs represent a promising step toward environmentally friendly remediation, further optimization is needed to reduce residual toxicity and assess long‐term and multispecies effects. The study also reinforces the utility of aquatic macrophytes as sentinel organisms in environmental monitoring and supports the development of more robust effluent management strategies that integrate both chemical and biological evaluations.

## 1 Introduction

Pollution of water bodies by tannery effluents poses a significant threat to environmental health and aquatic biodiversity [1]. As reported in various studies, among all industries, tanneries are considered some of the most polluting due to the massive use of toxic organic and inorganic compounds during leather processing [1, 2, 3, 4, 5]. Various ecotoxicological effects have been previously reported, including reductions in aquatic biodiversity, genetic mutations in aquatic organisms, disruptions in the life cycle of fish and invertebrates, and alterations in the biochemical and physiological processes of affected organisms [1, 6, 7, 8, 9, 10].

In plants, especially aquatic ones, previous investigations have demonstrated the adverse effects of untreated tannery effluents on various species. For example, the study conducted by Tejada‐Meza et al. [11] revealed that the release of untreated tannery effluents in Arequipa, Peru, caused a significant reduction in the number of fronds, wet weight, and dry weight of *Lemna minor* , as well as high mortality in *Daphnia magna* and *Physa venustula*, indicating the need for treatment of these effluents to prevent severe ecological impacts. De Sousa et al. [12] also observed that *Allium cepa* cultivated in water containing untreated tannery effluent reduced the mitotic index and chromosomal aberrations, demonstrating cytotoxic effects. Furthermore, Gupta [13], aiming to assess the bioaccumulation of Cr (VI) in *Pistia stratiotes* , noted a significant reduction in chlorophyll and protein content associated with high accumulation of Cr in the roots and stems, while Roy et al. [14] reported root growth inhibition and significant reductions in frond number, protein, and chlorophyll content in *A. cepa* and *L. minor* , as well as chlorosis and tissue necrosis in *Nostoc muscorum (cyanobacterium)*. Lal [15] found that *L. minor* exposed to different concentrations of tannery effluent exhibited significant biochemical changes, including increased levels of starch and soluble sugars at low concentrations but a sharp decrease at higher concentrations, along with variations in acid phosphatase activity. Thus, these studies provide substantial evidence supporting the hypothesis that the chemical components of untreated tannery effluents (both inorganic and organic) can induce various alterations in aquatic plants.

In this context, treating tannery effluents is crucial to reduce the load of toxic pollutants before they are discharged into the environment. According to studies reviewed by Bhardwaj et al. [2], various treatment methods have been proposed, including coagulation/flocculation, advanced oxidation processes (AOPs), biological treatment, membrane separation processes, and adsorption. However, these methods have several limitations, including the generation of large amounts of sludge, high operational costs, and complexity, which often render them unsustainable. This is why recent studies have explored the potential of hybrid and integrated technologies that combine different methods to improve efficiency and reduce costs, showing promising results in pollutant removal and economic feasibility. Furthermore, the sustainability of these methods can be enhanced by using abundant and natural materials, such as adsorbents derived from agro‐industrial waste and biomass. For example, the use of activated carbon produced from leather and cattle hair waste, as well as the application of natural coagulants like plant extracts, has shown significant potential in improving treatment efficiency and reducing environmental impact [16, 17]. Other examples include studies by Hashem et al. [18], Ameha et al. [19], Fseha et al. [20], Akter et al. [21], and Areti et al. [22], which evaluated the use of thermally activated adsorbents derived from kitchen waste biomass, banana peels, natural clay‐based materials, fish scales, and a mixture of banana peel and corn cob activated carbon, respectively, for treating tannery effluents. These studies have demonstrated the effectiveness of natural materials in pollutant removal, promoting sustainable and cost‐effective approaches that contribute to the circular economy and reduce dependence on synthetic compounds.

Obviously, in addition to the direct benefits of pollutant removal, treating effluents with advanced materials can significantly influence the ecological and physiological responses of exposed organisms, as demonstrated by the evidence observed by Bhattacharya et al. [23], Ashraf et al. [24], and Narayanan [25], who used different experimental models to assess the effects of treated effluents. Bhattacharya et al. [23] used the snail *Pila globosa* to compare the toxic impacts of untreated and membrane‐treated tannery effluents. The authors observed significant changes in biochemical parameters, such as increased activity of various antioxidant enzymes, DNA damage, and histological alterations in gonadal and mantle tissues. However, treatment of the effluents by microfiltration and reverse osmosis resulted in a significant reduction in toxicity. Ashraf et al. [24] assessed the toxic effects of tannery effluent on *Ctenopharyngodon idella* , revealing high acute toxicity and mortality due to the presence of high concentrations of salts and metals, and demonstrated that treatment with vegetated constructed wetlands significantly reduced toxicity. Meanwhile, Narayanan [25] investigated the use of metal‐tolerant fungal biomass to remove pollutants from tannery effluents, observing a significant reduction in toxicity in in vitro studies using *Vigna radiata* and *Artemia franciscana* .

On the other hand, Aguilar‐Ascon et al. [26] investigated the toxicity of effluents treated by electrocoagulation and ozonation using bioassays with *Lactuca sativa L*. seeds. The results showed that, despite the treatment, the effluents retained significant toxicity levels. Electrocoagulation removed 92% of total suspended solids (TSS) and 10% of chemical oxygen demand (COD), while ozonation reduced COD by 18%. However, the persistence of recalcitrant compounds resulted in continued adverse effects, underscoring the need to enhance treatment techniques to eliminate toxic pollutants. This example highlights the need to develop new treatment technologies and conduct ecotoxicological assessments following any treatment, as there is currently no consensus on the ecological benefits of these treatments. Additionally, the potential effects of treated tannery effluents on aquatic plants require further understanding. *Salvinia auriculata* , for instance, is an effective floating macrophyte for the absorption and accumulation of heavy metals and contaminants [27, 28, 29, 30], and is widely used in ecotoxicological studies [31, 32, 33].

In this context, our study aimed to evaluate the potential physiological and biochemical effects of tannery effluents treated with mercerized microcrystalline cellulose particles (MCPs) using *S. auriculata* as a model system. Although species such as *Lemna minor* and *Pistia stratiotes* are frequently employed in ecotoxicological studies, *S. auriculata* remains underexplored despite its recognized phytoremediation capacity, rapid clonal propagation, and sensitivity to waterborne contaminants. Therefore, its use as a test organism in this study introduces novelty and expands the ecotoxicological framework for evaluating the environmental risks associated with treated industrial effluents. The effluents used were obtained from the study by Gomes et al. (2025), which investigated the effectiveness of MCPs as a sustainable adsorbent for removing chromium (Cr) from tannery effluents. At that time, the authors obtained commercial MCPs, subjected them to mercerization, and conducted adsorption tests under various experimental conditions, observing a maximum adsorption capacity of 28.17 mg/g for Cr. However, a significant gap in this study and many similar ones is the need to evaluate the potential ecotoxicity of the treated effluents. Therefore, we aimed to determine how the treated effluents affect the growth, photosynthetic rate, and oxidative and antioxidant responses of the plants. Additionally, Cr concentrations were measured after 15 days of exposure. We hypothesized that tannery effluents treated with mercerized microcrystalline cellulose particles (MCPs) would exhibit significantly reduced toxicity to *S. auriculata* when compared to raw effluents, particularly in terms of chlorophyll content, oxidative stress biomarkers, and root development. This expectation was based on the documented efficiency of MCPs in reducing bioavailable Cr concentrations, potentially mitigating oxidative and physiological stress in aquatic plants. By investigating these issues, we aim to contribute to the understanding of effluent treatment methods, thereby aiding in the development of more effective environmental management strategies and sustainable practices.

## 2 Material and Methods

### 2.1 Tannery Effluent and Applied Treatment

The tannery effluent used in this study was the same one investigated by Gomes et al. (2025), obtained from an industry in Inhumas, Goiás, Brazil, and characterized by high chromium (Cr) concentration from the “wet‐blue” process. For the treatment of the raw effluent, Gomes et al. (2025) used mercerized microcrystalline cellulose particles (MCPs) characterized by Raman spectroscopy and scanning electron microscopy (SEM). The adsorption tests, conducted under various experimental conditions (stirring rate, pH, temperature, effluent concentration, and MCP concentration), showed a maximum adsorption capacity of 28.17 mg/g for Cr, with optimal conditions of 400 rpm, pH 5.0, 35°C, and 25 mg/L of MCPs. Therefore, in the present study, we used the raw tannery effluent (untreated) and the effluent treated under the optimal conditions established by Gomes et al. (2025).

### 2.2 Model System and Experimental Design

Aquatic plants of the species *S. auriculata* were collected from a lentic environment located in a Permanent Preservation Area (APP, i.e., a naturally protected area with strict exploitation limits) in Rio Verde, GO, Brazil, and transported to the Applied Environmental Toxicology Laboratory at the Federal Institute of Goiás—Urutaí Campus (GO, Brazil). The plants were then cultivated in tanks containing Hoagland solution (Hoagland and Arnon 1944), which was renewed every 7 days, without aeration, in a protected environment (a greenhouse with shade cloth covered with transparent plastic). After several generations of clonal propagation (propagation, frond separation, new propagation), we selected the plants used in the experiment.

Having been standardized for uniformity in size and leaf and root appearance, complete *S. auriculata* plants were introduced into containers (dimensions: 5.5 cm height × 10 cm diameter) containing 100 mL of dechlorinated tap water and acclimated for 5 days before the introduction of effluents, as described in Wolff et al. [34] and Mustafa and Hayder [35]. Subsequently, the plants were assigned to different experimental groups, with six replicates per group, each replicate consisting of a complete *S. auriculata* plant, that is, composed of two floating fronds, a submerged structure acting as a “root” [36], and two apical shoots. The control group (DWC) comprised plants maintained in dechlorinated water free of effluent. In contrast, the “RTE‐0.3” and “RTE‐3.1%” groups were formed by plants cultivated in dechlorinated water containing raw tannery effluent diluted to 0.3% and 3.1%, respectively. The plants grown in water containing tannery effluent, recovered from treatment with MCPs and diluted to 0.3% and 3.1%, respectively, constituted the “TTE‐0.3” and “TTE‐3.1%” groups. The dechlorinated tap water used in all experimental groups was previously analyzed for chromium concentration, which was below the quantification limit of the analytical method employed. As this water was uniformly used across all treatments, any residual levels of nutrients or other potential contaminants were equally diluted in all groups, ensuring that they did not interfere with the experimental outcomes.

The plants were exposed to the treatments for 15 consecutive days in the previously mentioned protected environment, under natural conditions (light, humidity, temperature, and wind). The exposure was static, that is, without complete water renewal, with only the evaporated volume being replaced every 2 days to ensure a constant water volume throughout the experiment. To achieve this, a mark was made on the containers, and the evaporated volume was replenished up to the original mark, ensuring stable conditions for a more realistic assessment of the potential impacts of the effluents on the aquatic plants.

#### 2.2.1 Determination of Tannery Effluent Dilutions

The tannery effluent dilutions used in the experiment were based on predictive scenarios of discharge into lentic environments, such as lakes and ponds, as supported by previous studies [37, 38]. The 0.3% dilution represents a low‐level discharge condition, similar to the 0.2% estimated by Montalvão et al. [39], who considered tannery processing capacities and the dilution potential of receiving water bodies. This scenario may occur during periods of reduced industrial activity or when improved wastewater management practices are implemented. In contrast, the 3.1% dilution reflects a high‐discharge scenario typical of peak production periods, when effluent output increases substantially. Together, these concentrations simulate ecologically relevant and realistic exposure conditions, allowing for a comprehensive assessment of the potential impacts of both untreated and treated tannery effluents.

### 2.3 Toxicity Biomarkers

#### 2.3.1 Growth

At the end of the experiment, to assess the impact of tannery effluents on plant growth, we used two sensitive biomarkers that reflect the effects of the treatments: the clonal unit dispersion area and the length of the “roots.” The dispersion area (%) was determined by capturing digital images of the plants in each experimental replicate and analyzing them with ImageJ software, as done by Gomes et al. [32], allowing us to evaluate whether the exposures influenced the plants' expansion capacity. The length of the modified submerged leaf structures (roots) was measured similarly, using images with a reference scale and analyzed in ImageJ, which was crucial for inferring the plants' ability to absorb nutrients and respond to environmental stresses.

#### 2.3.2 Photosynthetic Activity

To evaluate the potential impact of the effluents on the plants' photosynthetic activity, at the end of the experiment, fronds were collected for the determination of chlorophyll *a*, chlorophyll *b*, and total chlorophyll (*a* + *b*) levels, using procedures detailed in Gomes et al. [32], which were adapted from the protocols of Arnon [40] and Kumari et al. [41]. For each experimental replicate, two intermediate fronds (neither young nor senescent) were randomly collected from two of the three complete plants, totaling 12 frond samples per group. The selection of intermediate fronds aimed to minimize extreme phenological variations, ensuring that the samples represent a uniform developmental stage. The results were expressed as “mg of chlorophyll/g frond.” Additionally, we presented the ratio of chlorophyll *a* to chlorophyll *b* concentrations to identify potential changes in the relative proportion of these chlorophylls, which may indicate alterations in the plants' physiological state and the efficiency of the photosynthetic apparatus in response to effluent treatments.

#### 2.3.3 Toxicity Biomarkers

##### 2.3.3.1 Sample Collection and Initial Processing

To assess the potential effects of the exposures on the oxidative and antioxidative response of the plants, we randomly collected intermediate fronds (1 to 2) from one of the three complete plants per replicate, totaling eight frond samples per group. The samples were then macerated in phosphate‐buffered saline (PBS, pH 7.2, at 4°C) using a cell disruptor, followed by centrifugation at 13 000 rpm for 10 min at 4°C, with a collection of the supernatants. The supernatants were filtered through syringe filters with 0.45 μm pores and stored at −80°C until analysis. It is important to note that all procedures were carried out in a dim environment to avoid the effect of light on oxidative and antioxidant reactions.

##### 2.3.3.2 Oxidative Stress Biomarkers and Antioxidant Activity

To assess oxidative stress levels, the total levels of reactive oxygen species (ROS) were estimated using the protocol described by Gomes et al. [42], and the levels of malondialdehyde (MDA), a byproduct of lipid peroxidation (LPO) and a biomarker of oxidative stress, were quantified based on the procedures described by Sachett et al. [43]. Additionally, to correlate oxidative stress results with the antioxidant response, we evaluated the activities of the enzymes superoxide dismutase (SOD) and catalase (CAT), which are considered the primary lines of antioxidant defense in organisms [44]. While SOD activity was assessed using an assay based on the auto‐oxidation of pyrogallol, as previously described [45], CAT activity was determined using the method described by Hadwan and Abed [46], which is based on the reaction of undecomposed hydrogen peroxide with ammonium molybdate, producing a yellowish color. Additionally, the antioxidant capacity of the plants was estimated using the DPPH (2,2‐diphenyl‐1‐picryl‐hydrazyl‐hydrate) free radical method, according to the methodology described by Brand‐Williams et al. [47]. This method allows us to assess the total capacity of the plants to neutralize free radicals, including not only the activity of the antioxidant enzymes SOD and CAT but also the contribution of other antioxidant compounds present in plant tissues, such as flavonoids, phenols, and other bioactive molecules that protect against oxidative stress.

To evaluate the relationship between oxidative stress and antioxidant activity, we calculated the ratios between DPPH inhibition and SOD activity (DPPH inhibition/SOD activity), DPPH inhibition and CAT activity (DPPH inhibition/CAT activity), DPPH inhibition and ROS levels (DPPH inhibition/ROS levels), as well as the ratios between SOD and CAT activity (SOD/CAT activity) and CAT and SOD activity (CAT/SOD activity). These ratios provide a more detailed understanding of how specific antioxidant activities of the enzymes SOD and CAT, along with other antioxidant compounds measured by DPPH inhibition capacity, are related to oxidative stress levels represented by ROS. Analyzing these relationships is crucial for understanding the effectiveness of the plant antioxidant mechanisms in neutralizing oxidative stress induced by the experimental conditions.

##### 2.3.3.3 Nitrosative Stress Biomarkers

Considering that the production of nitrogen free radicals can cause significant damage to plant cells and tissues and is closely related to antioxidant activity [48], nitric oxide levels were assessed in the plants via an indirect measure of nitrite production using the procedures detailed in Gomes et al. [42], adapted from Bryan and Grisham [49]. The assessment of nitric oxide levels, along with oxidative stress biomarkers and the activity of the antioxidant enzymes SOD and CAT, provides a comprehensive view of the plants' responses to environmental stresses, allowing for a better understanding of the antioxidant and nitrosative defense mechanisms.

##### 2.3.3.4 Total Protein

Considering that the samples were not weighed before processing to avoid exposure to light, temperature variations, and other factors that could influence the results, the total protein levels of all samples were determined using the Bradford method [50] to ensure measurement accuracy. Thus, the results of all biochemical analyses were normalized and expressed as “unit of each biomarker/μg of protein.” It is also noted that all procedures for biochemical analyses and evaluating the plants' photosynthetic activity were strictly followed according to quality standards. Blank samples and negative controls were used to ensure measurement accuracy, and calibrated equipment and analytical grade reagents were employed to minimize technical variability. Furthermore, the analyses were performed in triplicate to ensure reproducibility, and the data were statistically validated to confirm the significance of the findings.

### 2.4 Determination of Chromium Concentration

Since chromium is one of the main contaminants in tannery effluents and the treatment applied to the effluents provided by Gomes et al. (2025) specifically targeted the removal of this metal, its concentrations were evaluated in the samples of fronds and “roots” remaining after material collection for the previously described analyses. The digestion was performed on the remaining leaves and “roots”; the leaves were removed for the analyses, and the remaining material was dried in an oven at 70°C until a constant weight was achieved. Then, the samples were processed based on Chagas et al. [37], with some modifications. Briefly, the samples were placed in digestion tubes with 1 mL of perchloric acid (P.A.) and left overnight. After pre‐digestion, the temperature was gradually increased to 220°C until the material was completely digested. Subsequently, the tube was cooled to room temperature (25°C), and 1 mL of 5% nitric acid was added, adjusting the volume to 5 mL. After complete digestion, the extracts were filtered and diluted in deionized water.

The chromium concentrations were then determined using flame atomic absorption spectroscopy (AAS), as described in Ajlec et al. [51]. Certified chromium standards were used for equipment calibration to ensure the accuracy and reliability of measurements. Additionally, blanks and internal controls were included in each analysis batch to monitor the accuracy and precision of the analytical procedures. Quality control measures included the analysis of duplicate samples and the verification of the recovery of standards added to the samples. Spectrophotometer calibration was performed using calibration curves constructed with at least five chromium standard concentrations. The results were expressed in milligrams of chromium per gram of dry weight of the samples, allowing for an accurate assessment of the treatment's efficiency in removing chromium from the effluents and its impact on bioaccumulation by the plants. All analyses were performed by a certified commercial laboratory accredited by regulatory agencies under ISO/IEC 17025 standards, ensuring full traceability, adherence to rigorous quality control protocols, and reliability of the analytical results.

### 2.5 Statistical Analyses

The statistical analyses conducted in this study aimed to evaluate the significance of the obtained results and to identify patterns and relationships between the investigated variables. The statistical procedures included Analysis of Variance (ANOVA), Kruskal–Wallis test, Principal Component Analysis (PCA), correlation analysis followed by linear regression, and hierarchical clustering analysis (Cluster Analysis) using Ward's method with Euclidean distance. We used PrismGraph software for ANOVA, Kruskal–Wallis test, PCA, correlation and linear regression analysis, and PAST (PAleontological STatistics) software for Cluster analysis, with a significance level of 5% (*p* < 0.05) for all analyses.

The ANOVA was used to compare the means of different experimental groups. The choice of statistical test for each biomarker was based on the specific distributional characteristics of the data. Before ANOVA, we verified the normality and homogeneity of variances assumptions using the Shapiro–Wilk and Levene tests, respectively. If both assumptions were met, one‐way ANOVA followed by Tukey's post hoc test was applied. The non‐parametric Kruskal–Wallis test was employed for data that violated these assumptions, followed by Dunn's post hoc test with Bonferroni correction for multiple comparisons. The decision regarding the use of parametric or non‐parametric tests is explicitly indicated in each figure, together with the respective test statistics and *p* values, ensuring full transparency and reproducibility of the statistical procedures. Principal Component Analysis (PCA) was performed to reduce data dimensionality and identify patterns of variability. The data were transformed using log10 and normalized, as this transformation helps to manage variations in data magnitudes and improves distribution for subsequent analyses. Additionally, outliers were removed using the Grubbs test. The correlation matrix was used for PCA, which is suitable for data with different measurement units. Principal components were selected based on eigenvalues greater than 1 (Kaiser criterion) and by inspecting the scree plot. We interpreted the factor loadings to identify the most influential variables in each principal component.

Hierarchical Cluster Analysis was used to identify similarity groups among the experimental groups, complementing PCA by indicating clustering patterns and proximity relationships between the groups. We chose the Ward method because it minimizes cluster variance and uses Euclidean distance to measure group similarity. The resulting dendrogram was analyzed to determine the number of clusters and interpret the similarity relationships. Additionally, correlation analysis was performed to identify significant relationships between variables using Spearman and Pearson correlation coefficients, as appropriate. Pearson correlation was used for parametric data, while Spearman correlation was applied for non‐parametric data. If a significant correlation (*p* < 0.05) was found, we proceeded with linear regression to quantify the relationship and predict the values of one variable based on another.

## 3 Results

**FIGURE 1:**
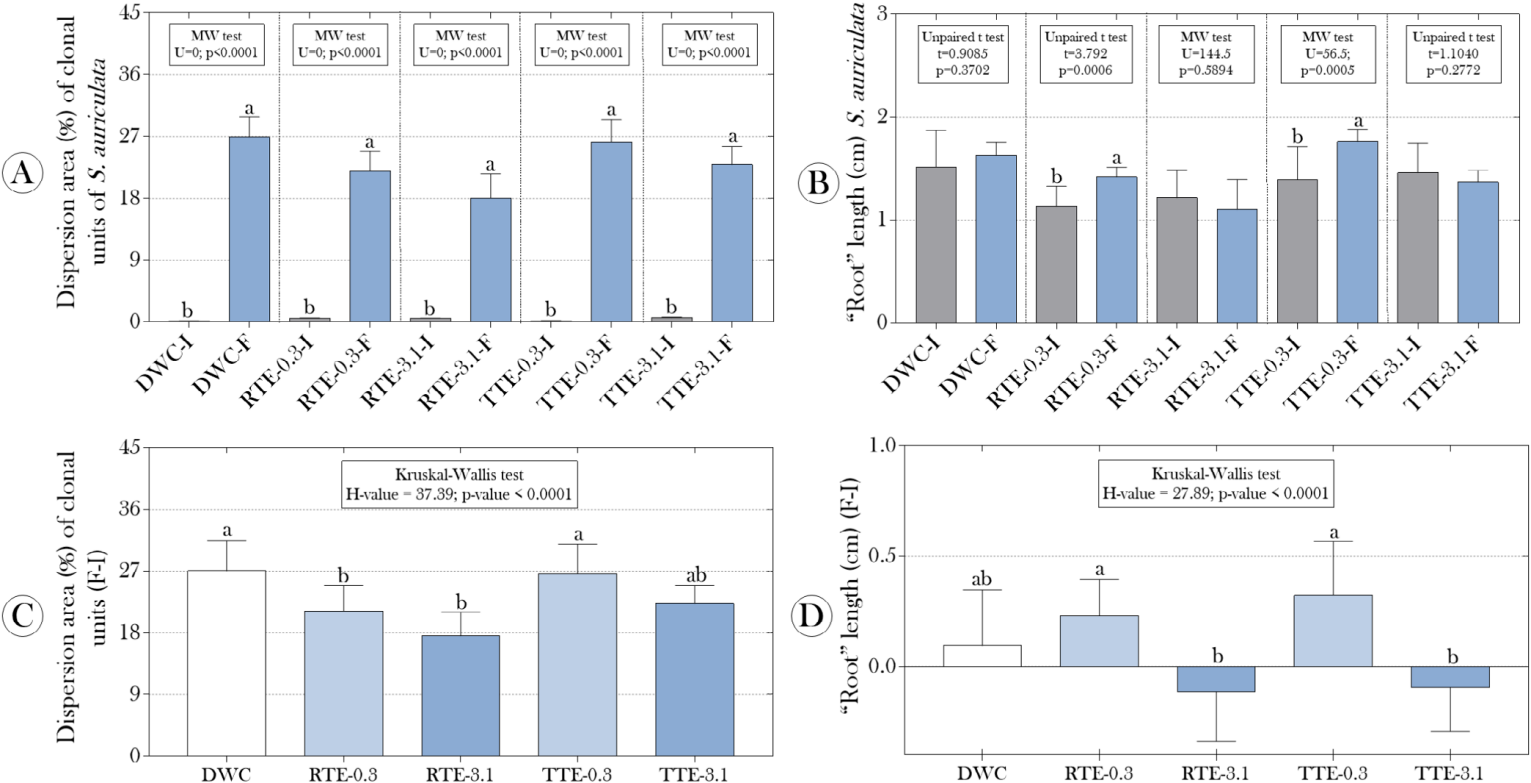
Impact of raw and cellulose mercerized microcrystalline particle (CMP)‐treated tannery effluents on the growth and clonal dispersion of *Salvinia auriculata* after 15 days of exposure. The figure presents the effects of raw tannery effluents (RTE) and tannery effluents treated with CMPs (TTE) at two concentrations (0.3% and 3.1%) on the dispersion area of clonal units (A and C) and root length (B and D) of *S. auriculata* . Parametric data are presented as mean + standard deviation (A and B), and non‐parametric data are shown as median and interquartile range (C and D). Statistical test summaries are shown above the bars and include: *t*‐value (Student's *t*‐test), MW (Mann–Whitney *U* test), *H*‐value (Kruskal–Wallis test), and *U*‐value (Mann–Whitney statistic). Different lowercase letters indicate statistically significant differences between groups (*p* < 0.05), determined using Tukey's, Dunn's, or Mann–Whitney post hoc tests, depending on data distribution and test applied.

Initially, our results did not show significant differences in the spread area of the clonal units of *S. auriculata* measured at the experiment's beginning and end, regardless of the treatment applied (Figure 1A). However, the exposures affected the growth of the “roots,” inferred from root length. As shown in Figure 1B, root growth (between the start and end of the exposures) was observed only in plants exposed to the effluents diluted to 0.3% (RTE‐0.3 and TTE‐0.3%, respectively). Furthermore, when analyzing the delta of the spread area, we found less dispersion in plants exposed to raw effluents (RTE‐0.3 and RTE‐3.1 groups) (Figure 1C). For the delta of root length, negative values were reported for plants exposed to the higher concentration of effluents, whether raw or previously treated with CMPs (Figure 1D).

**FIGURE 2:**
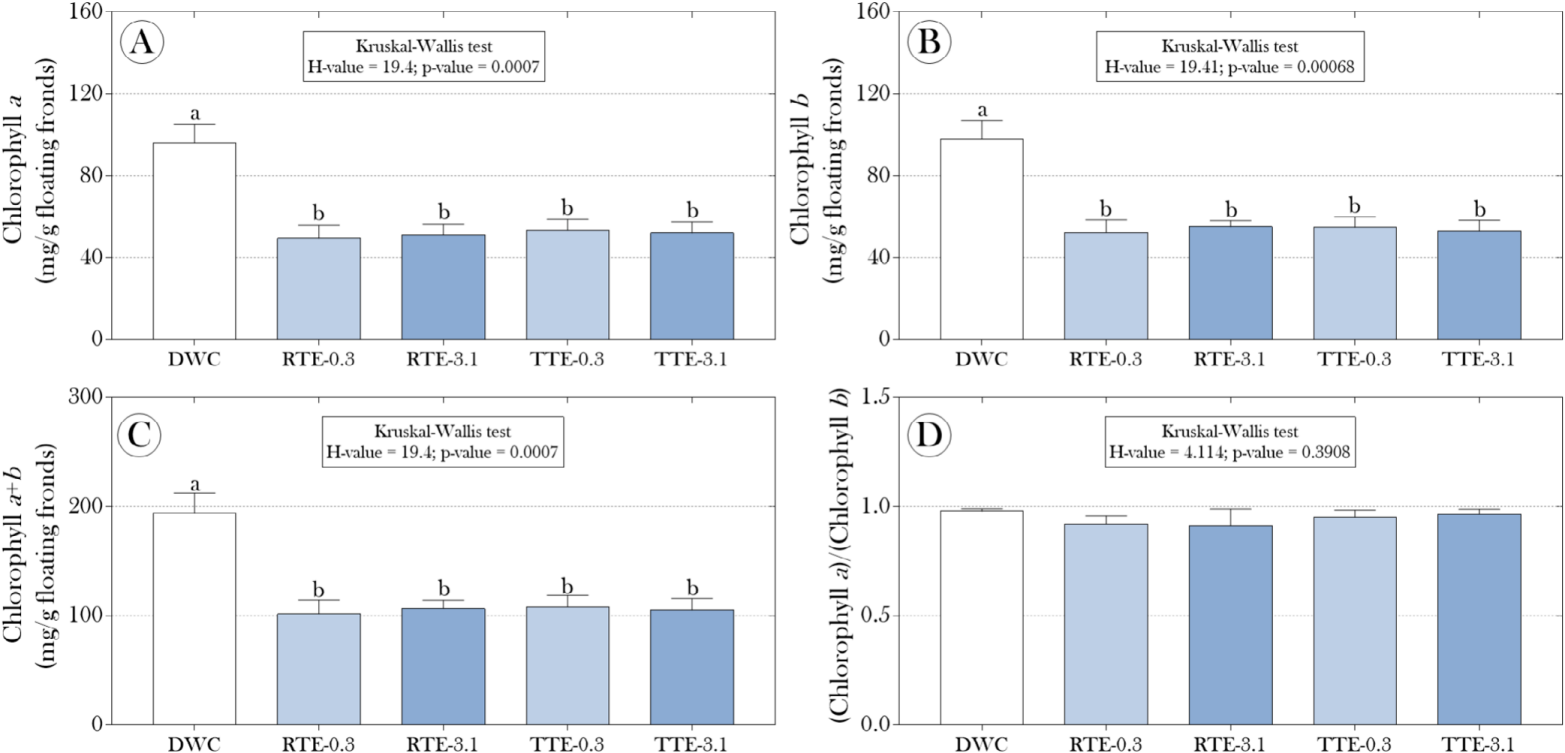
Impact of raw and treated tannery effluents with mercerized microcrystalline cellulose (CMPs) on the levels of (A) chlorophyll *a*, (B) chlorophyll *b*, and (C) total chlorophyll (*a* + *b*) in floating fronds of *Salvinia auriculata* after 15 days of exposure, as well as (D) the ratio between chlorophyll *a* and *b* levels. Parametric data are presented as mean + standard deviation, while non‐parametric data are presented as median and interquartile range. Statistical summaries are presented above the bars. Distinct lowercase letters indicate significant differences between experimental groups at a 95% significance level (*p* < 0.05).

Regarding the photosynthetic activity of the plants, our results showed lower levels of chlorophyll *a* (Figure 2A), chlorophyll *b* (Figure 2B), and total chlorophyll (*a* + *b*) (Figure 2C) in the plants exposed to the effluents, regardless of type and dilution. However, the evaluation of the ratio between chlorophyll *a* and chlorophyll *b* did not reveal significant differences between experimental groups (Figure 2D), indicating that although the absolute levels were affected, the proportion between the types of chlorophyll was not altered by the exposures.

**FIGURE 3:**
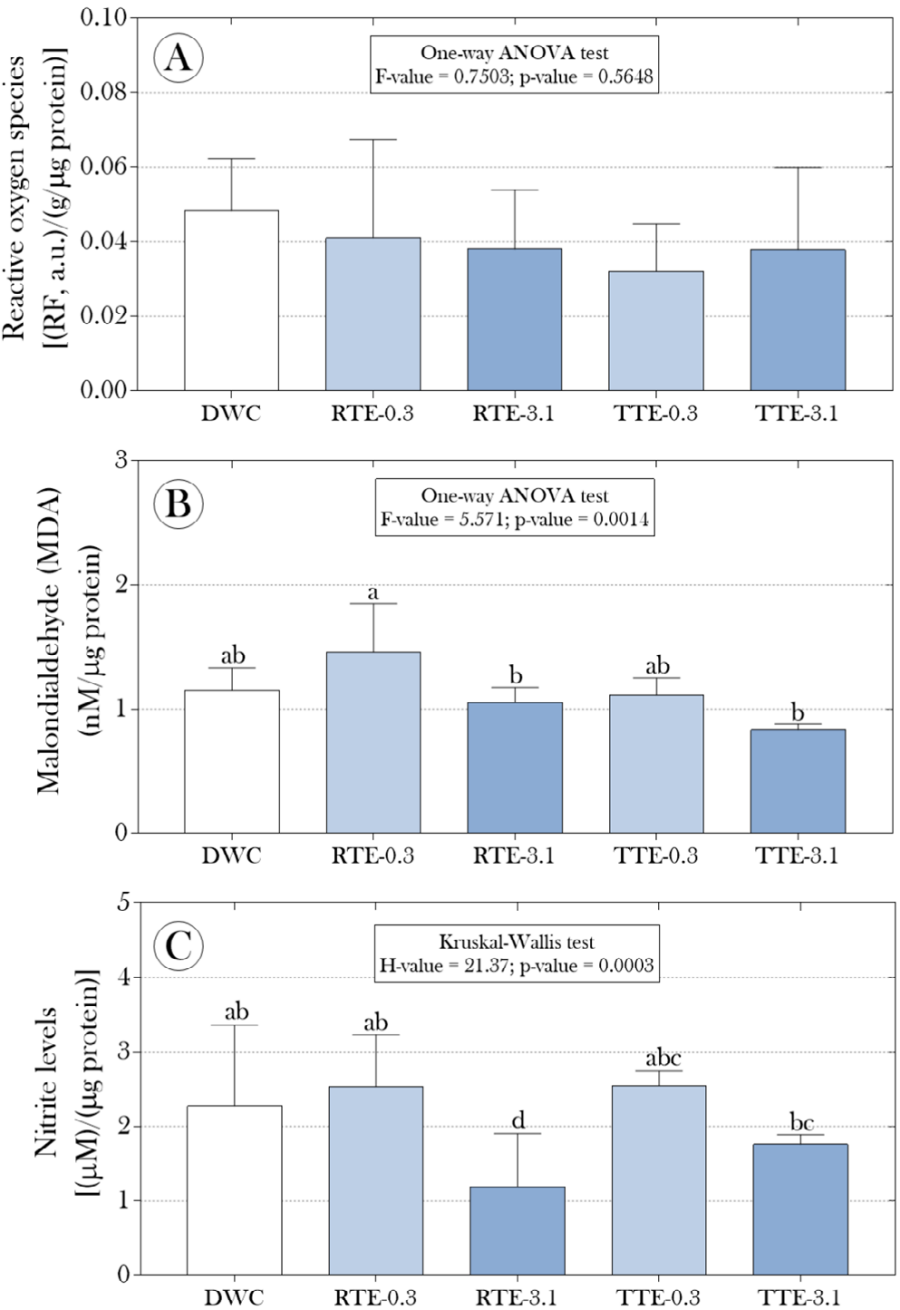
Impact of raw and microcrystalline cellulose mercerized (CMPs) tannery effluents on the production of (A) reactive oxygen species (ROS), (B) malondialdehyde (MDA), and (C) nitrite in floating fronds of *Salvinia auriculata* after 15 days of exposure. Parametric data are presented as mean + standard deviation, while non‐parametric data are presented as median and interquartile range. Statistical summaries are shown above the bars. Distinct lowercase letters indicate significant differences between experimental groups at a 95% significance level (*p* < 0.05).

**FIGURE 4:**
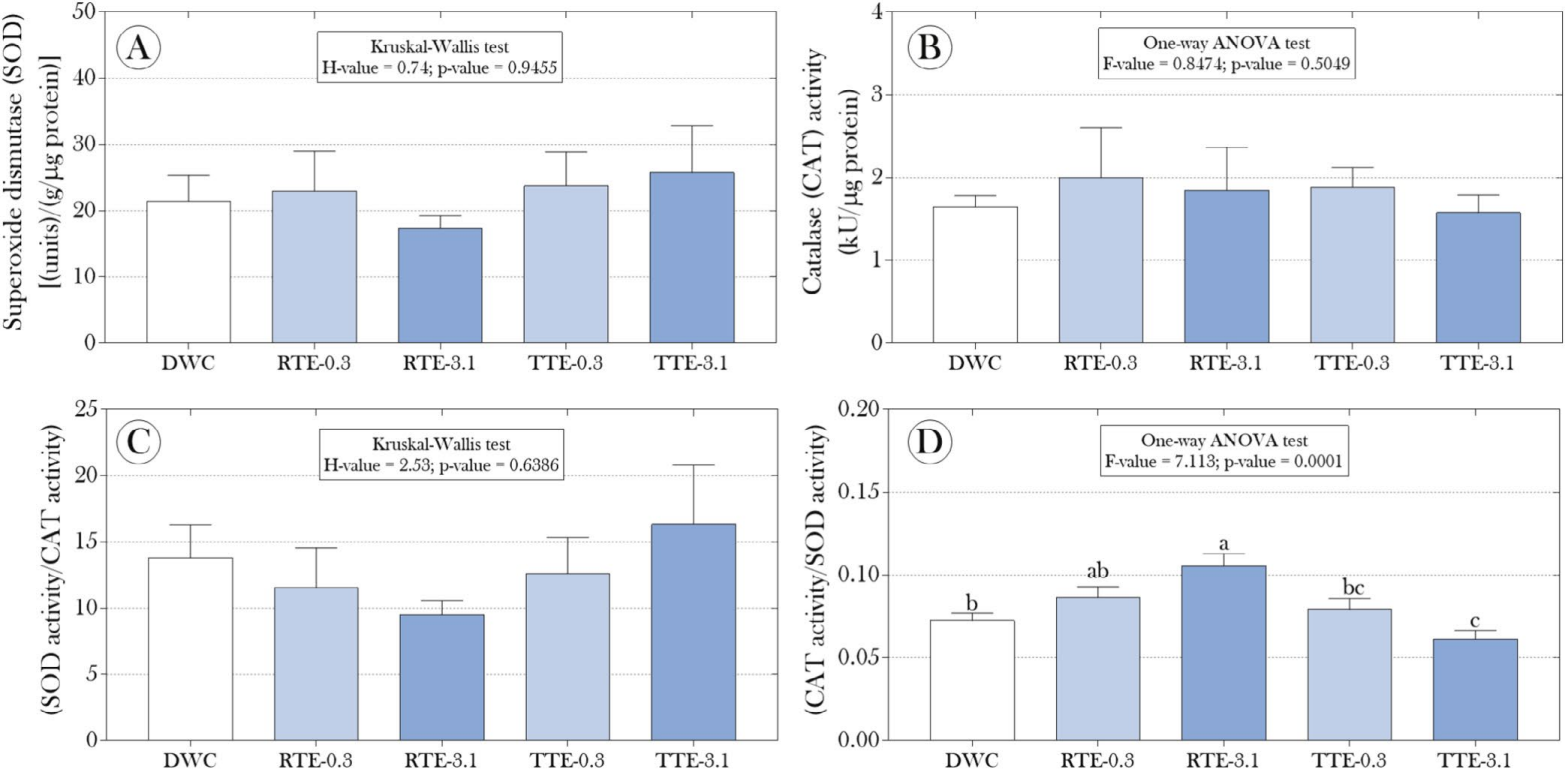
Impact of raw and microcrystalline cellulose mercerized (CMPs) tannery effluents on the activity of (A) superoxide dismutase (SOD) and (B) catalase in floating fronds of *Salvinia auriculata* after 15 days of exposure, as well as (C, D) the ratios between the activities of these enzymes. Parametric data are presented as mean + standard deviation, while non‐parametric data are presented as median and interquartile range. Statistical summaries are shown above the bars. Distinct lowercase letters indicate significant differences between experimental groups at a 95% significance level (*p* < 0.05).

Additionally, assuming that exposure to the effluents might induce a redox imbalance, we assessed ROS and MDA levels, as well as the activity of the SOD and CAT enzymes in *S. auriculata* fronds. However, significant differences in these biomarkers were not observed between the plants exposed to the effluents and those in the DWC group (Figures 3A,B and 4A,B). On the other hand, although the ratio between SOD and CAT activity was not affected by the exposures (Figure 4C), for the inverse of this relationship (i.e., CAT/SOD activity), we observed higher values in the RTE‐3.1 group and lower values in the TTE‐3.1 group compared to the non‐exposed plants (Figure 4D). While the difference between the RTE‐3.1 and DWC groups was approximately 45.8%, a reduction of about 18% was observed in the TTE‐3.1 group.

**FIGURE 5:**
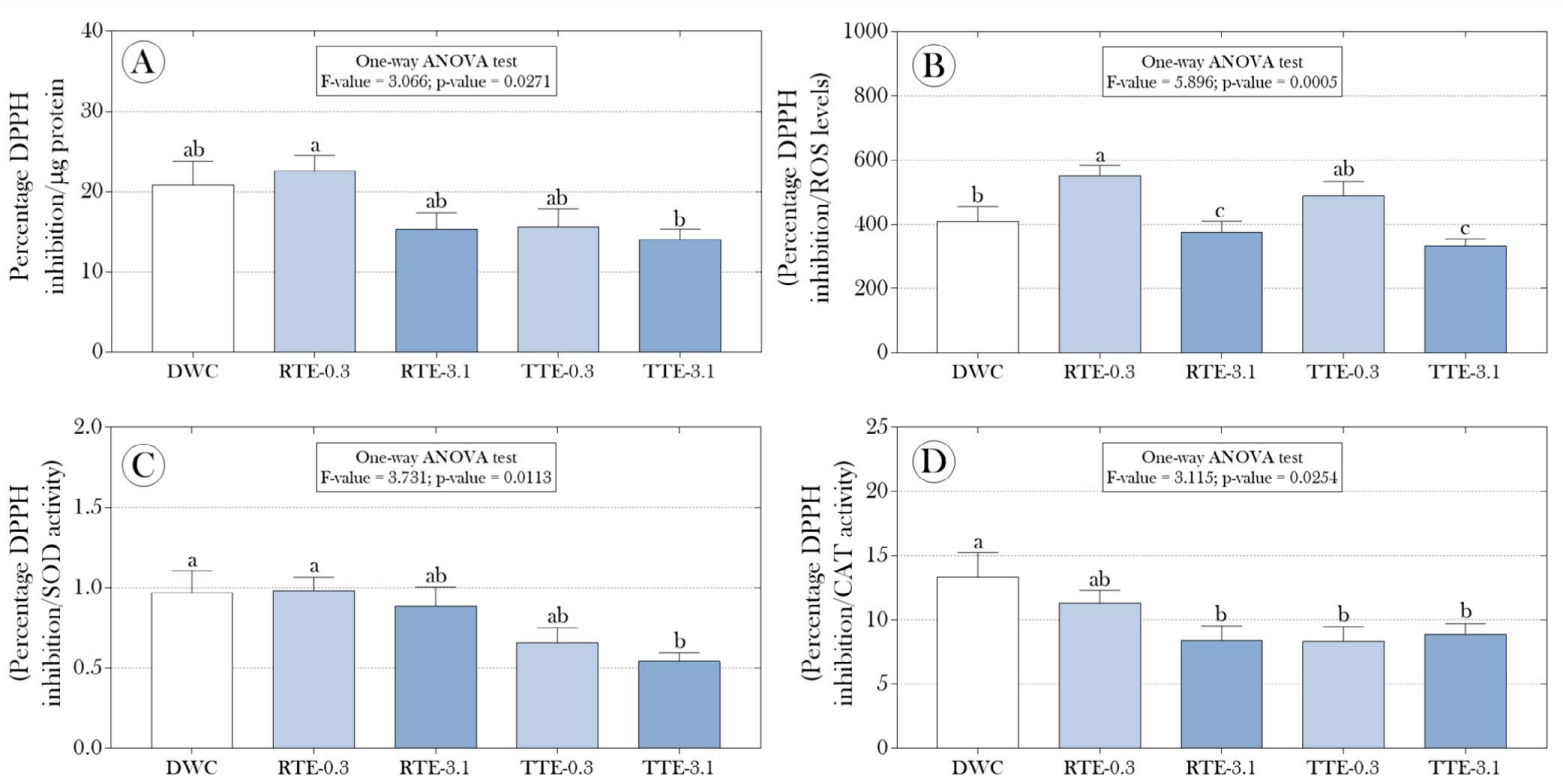
Impact of raw and treated (with mercerized microcrystalline cellulose, CMPs) tannery effluents on the percentage inhibition of the DPPH radical in floating fronds of *Salvinia auriculata* after 15 days of exposure and its relationships with the production of reactive oxygen species (ROS) (B) and the activity of the enzymes superoxide dismutase (SOD) (C) and catalase (CAT) (D). Parametric data are presented as mean + standard deviation, while non‐parametric data are presented as median and interquartile range. Statistical summaries are shown above the bars. Distinct lowercase letters indicate significant differences between experimental groups at a 95% significance level (*p* < 0.05).

In addition to the activities of the SOD and CAT enzymes, considered the primary lines of antioxidant defense, we evaluated the percentage inhibition of the DPPH radical, assuming that exposure to effluents could alter the overall antioxidant capacity of the plants, which includes not only SOD and CAT but also other antioxidants (both enzymatic and non‐enzymatic). Our results revealed that, although the experimental groups did not differ significantly regarding overall antioxidant capacity (Figure 5A), exploring the ratios between different biomarkers showed significant effects associated with effluent exposure. In the exposure to raw effluent diluted to 3.1% (RTE‐3.1 group), the ratio between the percentage inhibition of the DPPH radical and ROS levels was significantly lower than that observed in non‐exposed plants (approximately 10% reduction). An even more pronounced reduction of 22.4% was observed in the group exposed to treated effluent (TTE‐3.1) (Figure 5B). On the other hand, in plants exposed to raw effluent at the lower dilution (RTE‐0.3 group), a significant increase of 35.10% was observed compared to the DWC group (Figure 5B). Additionally, the ratios between DPPH inhibition and the activities of SOD and CAT enzymes were lower, especially in plants exposed to previously treated effluents (both diluted to 0.3% and 3.1%) (Figure 5C,D), suggesting a complex modulation of the antioxidant response beyond the simple activity of SOD and CAT. Regarding the potential induction of nitrosative stress by the exposures, Figure 3C shows that exposure to raw effluent diluted to 3.1% (RTE‐3.1 group) resulted in a significant suppression of nitrite production in the floating fronds of *S. auriculata* . Specifically, the average nitrite levels in the control group (DWC) were 2.27 μM/μg of protein. In contrast, in the RTE‐3.1 group, the average was 1.18 μM/μg of protein, representing an approximate reduction of 48%.

**FIGURE 6:**
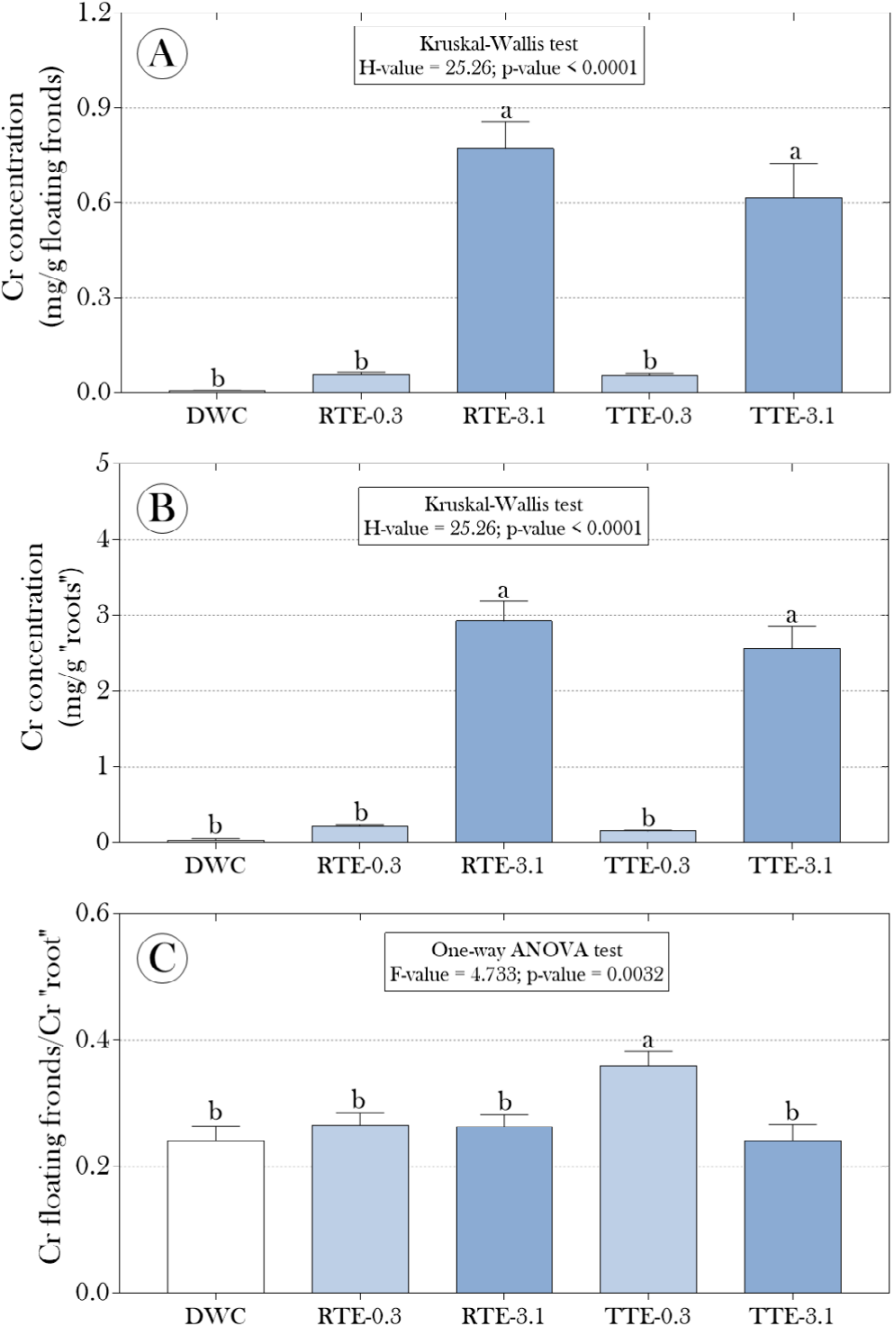
Chromium (Cr) concentrations in (A) floating fronds and (B) “roots” of *Salvinia auriculata* exposed to raw and treated tannery effluents with mercerized microcrystalline cellulose (CMPs) for 15 days. (C) The ratio between Cr concentrations in fronds and “roots.” Parametric data are presented as mean + standard deviation, while non‐parametric data are presented as median and interquartile range. Statistical summaries are shown above the bars. Distinct lowercase letters indicate significant differences between experimental groups at a 95% significance level (*p* < 0.05).

Furthermore, we quantified the Cr concentration in the floating fronds and “roots” of *S. auriculata* to assess the potential distribution and accumulation of this metal in different parts of the plant, assuming that the treatment of the effluent with CMPs was not able to remove 100% of the metal. As shown in Figure 6A,B, Cr concentrations were significantly higher in plants exposed to effluents diluted to 3.1% compared to the other experimental groups. Additionally, we observed that the ratio between Cr concentrations in the fronds and “roots” was significantly higher in plants exposed to the treated effluent diluted to 0.3% (Figure 6C), which supports the hypothesis that the metal distribution between different parts of the plant was altered by exposure to this type of effluent and dilution.

**FIGURE 7:**
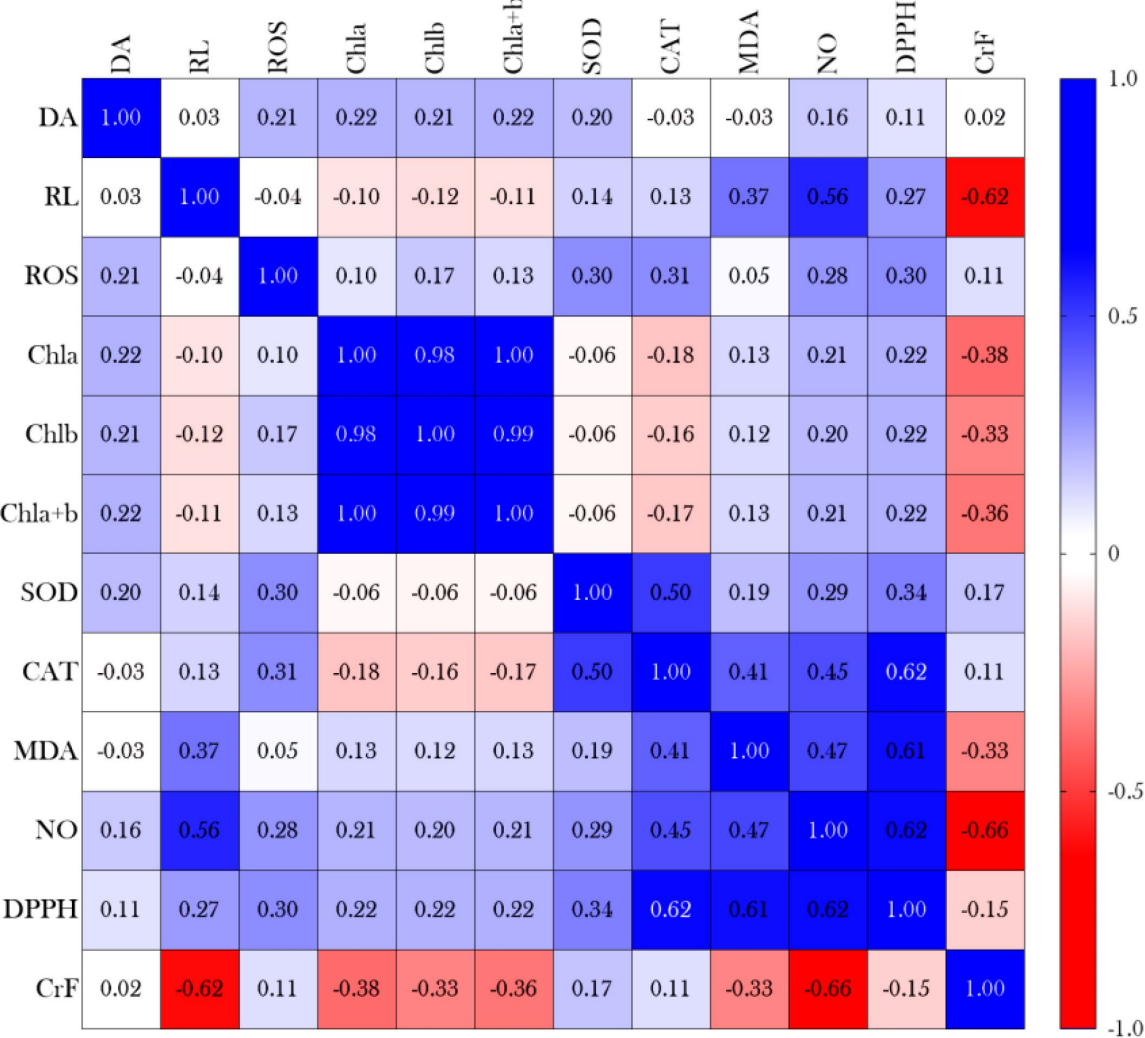
Heatmap representation of the correlation matrix showing the correlations between different physiological and biochemical biomarkers measured in *Salvinia auriculata* exposed to raw and mercerized microcrystalline cellulose‐treated (CMPs) tannery effluents for 15 days. Colors indicate the strength and direction of correlations, where values close to 1 (in blue) represent a strong positive correlation, values close to −1 (in red) represent a strong negative correlation, and values close to 0 indicate little or no correlation. CAT: catalase; Chl*a*, Chl*b*, and Chl*a* + *b*: chlorophyll *a*, *b*, and total (*a* + *b*); CrF: chromium concentration in floating fronds; DA: dispersion area of clonal units; DPPH: percentage inhibition of the DPPH radical; MDA: malondialdehyde; NO: nitric oxide (inferred from nitrite levels); RL: “root” length; ROS: reactive oxygen species; SOD: superoxide dismutase.

**FIGURE 8:**
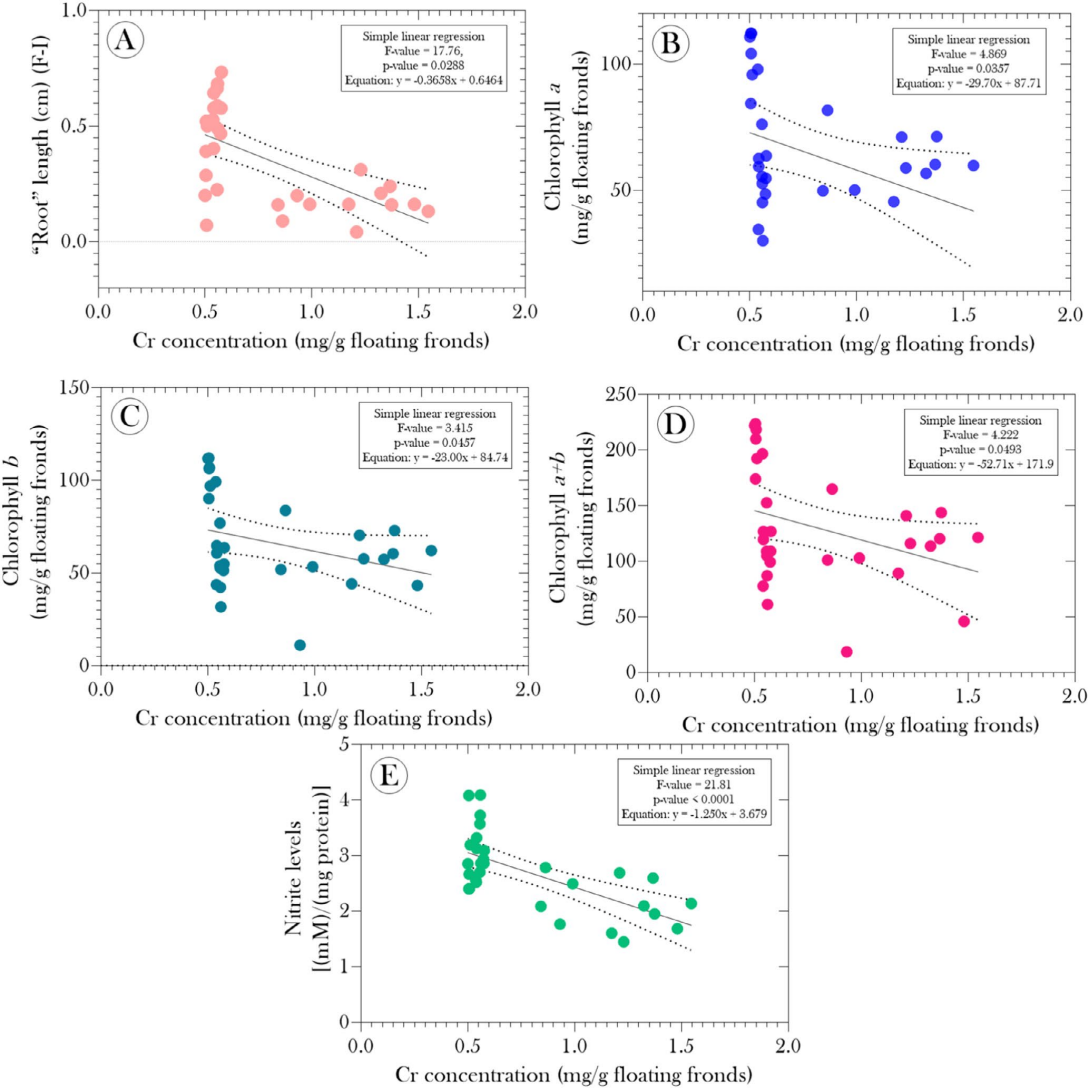
Simple linear regressions showing the relationship between chromium concentration in the floating fronds of *Salvinia auriculata* exposed to raw and mercerized microcrystalline cellulose‐treated (CMPs) tannery effluents for 15 days and different biomarkers: (A) “root” length (RL), (B) chlorophyll *a* concentration (Chl*a*), (C) chlorophyll *b* concentration (Chl*b*), (D) total chlorophyll (Chl*a* + Chl*b*), and (E) nitrite concentration. The solid lines represent the best‐fit line for the data, while the dashed lines indicate the 95% confidence bands around the best‐fit line.

In this regard, considering the higher Cr concentration in the fronds and its possible implications for the biochemical responses of *S. auriculata* , we performed a correlation analysis to identify potential relationships between Cr accumulation and the assessed biomarkers. Figure 7 shows a negative correlation between Cr concentration in the fronds (CrF) and the length of the “roots” (RL) (*r* = −0.62; *p* value = 0.0002), chlorophyll *a* content (Chl*a*) (*r* = −0.38; *p* value = 0.0357), chlorophyll *b* content (Chl*b*) (*r* = −0.33; *p* value = 0.0410), and total chlorophyll (Chl*a* + *b*) (*r* = −0.36; *p* value = 0.0493). Furthermore, we observed a strong negative correlation between CrF and nitrite levels (*r* = −0.66; *p* value < 0.0001), suggesting a possible interference of Cr in NO signaling. Linear regression results for these correlations confirm these negative associations, indicating that an increase in Cr concentration in the fronds is directly related to a significant decrease in the length of the “roots” (Figure 8A), as well as in chlorophyll concentrations (Figure 8B), chlorophyll *b* concentrations (Figure 8C), and total chlorophyll (Figure 8D). Additionally, linear regression between CrF and NO levels (Figure 8E) shows a clear trend of reduced nitrite production as Cr accumulation increases. These findings reinforce the idea that Cr accumulation in the fronds of *S. auriculata* may trigger adverse physiological responses impacting both plant growth and antioxidant capacity.

**FIGURE 9:**
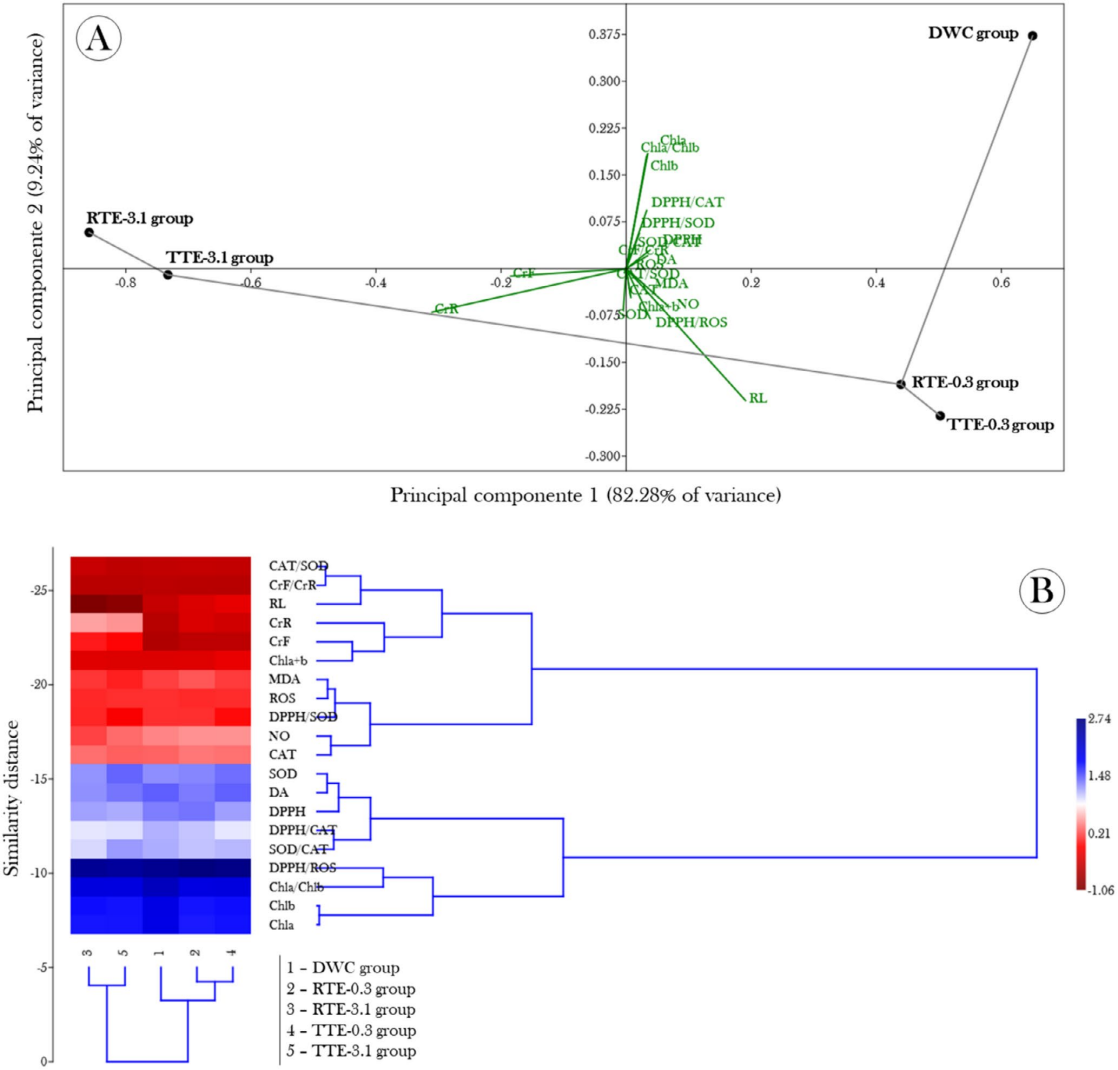
(A) Biplot of Principal Component Analysis (PCA) and (B) a dendrogram from hierarchical cluster analysis of the biomarkers assessed in *Salvinia auriculata* exposed to raw tannery effluents and treated with mercerized microcrystalline cellulose (CMPs) for 15 days. In (A), experimental groups are represented by black circles, and the lines connecting these circles can be interpreted as a minimum spanning tree, illustrating the proximity and relationships between the groups in the principal component space. Green vectors represent the biomarkers, indicating the direction and magnitude of each variable's influence on the separation of the groups along the two main components. In (B), the dendrogram, accompanied by a heatmap, displays the similarity between experimental groups (indicated by 1 to 5) and the relationships among biomarkers, highlighting the clusters formed based on Euclidean distances.

Based on the results obtained in our study, we explored the interrelationships between the assessed biomarkers and the different experimental groups using PCA and Cluster Analysis, aiming to identify variability patterns and possible groupings that explained the observed intergroup differences. PCA revealed that the first two principal components (PC1 and PC2) together explained 94.53% of the total variance in the data, with 85.28% attributed to PC1 and 9.25% to PC2. In Figure 9A, a clear separation of groups is observed along PC1, where the groups exposed to raw and treated effluents at 3.1% dilution (RTE‐3.1 and TTE‐3.1) are positioned negatively, while the control group (DWC) and groups exposed to effluent diluted to 0.3% (RTE‐0.3 and TTE‐0.3) are distributed positively. This separation suggests that PC1 is strongly associated with chromium concentration in the fronds (CrF) and the ratio between Cr in fronds and “roots” (CrF/CrR), which showed high negative loadings, confirming previous findings from ANOVA or Kruskal–Wallis tests where Cr bioaccumulation was crucial for distinguishing between groups. Additionally, the significant correlations observed earlier between CrF and biochemical biomarkers, such as chlorophyll and nitric oxide levels, were corroborated by PCA, which showed that variables associated with photosynthesis (Chl*a*, Chl*b*) and “root” length (RL) significantly contributed to the variability explained by PC2. These findings, along with the results from linear regression analyses, reinforce the idea that Cr accumulation in fronds is a determining factor in the observed biochemical changes, particularly regarding photosynthesis and oxidative stress.

Cluster analysis, as illustrated by the dendrogram and heatmap presented in Figure 9B, complements these findings by revealing clear groupings among the experimental groups and biochemical variables. It can be seen that the RTE‐3.1 and TTE‐3.1 groups, which were highlighted in PCA for their distinct biochemical characteristics, form a separate cluster from the other groups. This indicates that the biochemical responses of these groups are significantly different from those of the control group (DWC) and the groups exposed to effluents diluted to 0.3%. Notably, variables related to oxidative stress and photosynthesis, such as Cr concentration in the fronds, “root” length, and the ratios between chlorophyll levels, cluster distinctly from variables associated with antioxidant defenses, such as SOD and CAT activities and overall antioxidant capacity (DPPH). This pattern suggests a dissociation between Cr bioaccumulation and antioxidant response, with Cr accumulation predominantly affecting photosynthesis and plant growth, while antioxidant defenses appear to be more modulated by specific effluent concentrations and treatments. Thus, Cluster Analysis not only confirms the distinctions observed in PCA but also provides an integrated and hierarchical view of the relationships between biochemical variables and treatment groups, highlighting the complex interactions governing the physiological responses of *S. auriculata* to tannery effluent exposure.

## 4 Discussion

The contamination of aquatic ecosystems by industrial effluents, especially those from tanneries, poses a significant environmental threat due to the presence of heavy metals, such as chromium (Cr), and a variety of toxic organic compounds [1]. Cr, in particular, is widely recognized for its high toxicity [52], bioaccumulation potential [53], and environmental persistence [54], raising concerns about the health of aquatic ecosystems and food safety. Despite technological advances in effluent treatment, effectively removing Cr and other contaminants or pollutants remains challenging. The mercerization of microcrystalline cellulose (CMPs) was proposed by Gomes et al. [42] as an innovative approach for the adsorption of this metal, leveraging the unique properties of cellulose, such as its high ion exchange capacity and biodegradability. However, the effectiveness of this method under exposure conditions and its ecotoxicological impacts on aquatic organisms had not been sufficiently explored. Therefore, our study aimed to fill this gap by assessing the potential impact of tannery effluents treated with mercerized CMPs using *S. auriculata* as a model organism. The main findings indicate that although treatment with mercerized CMPs partially mitigated toxicity, plant growth, photosynthesis, and antioxidant responses were significantly affected, reflecting a complex interaction between Cr accumulation and other potential contaminants not quantified in the effluents.

Initially, we demonstrated that exposure to tannery effluents, even those previously treated with CMPs, significantly impacted both the growth and photosynthetic activity of the evaluated plants. The analysis of clonal unit dispersion area and “root” length (Figure 1) revealed a marked reduction in the groups exposed to both raw and treated effluents, especially at higher concentrations (RTE‐3.1 and TTE‐3.1). These findings suggest that the bioaccumulation of Cr in the fronds and roots critically interfered with root development, possibly through direct interference with metabolic processes and ionic homeostasis. Although Cr, in its hexavalent form (Cr VI), is known to generate reactive oxygen species (ROS) during its reduction to Cr III [55], we did not observe a significant increase in ROS levels in the exposed groups (Figure 3A), suggesting that Cr may have acted through other mechanisms. A plausible explanation is that Cr might have directly competed with other essential metal ions, such as magnesium and calcium, which are vital for cellular structural stability and signaling. Competition for magnesium, in particular, could have impaired chlorophyll synthesis, leading to decreased photosynthetic efficiency, while interference with calcium may have compromised cell wall integrity and root growth [56, 57]. Moreover, chromium might have interacted with thiol groups in proteins, inactivating essential enzymes and disrupting cellular metabolism in ways that did not necessarily result in increased ROS levels but nonetheless compromised vital functions such as cell division and root elongation. These mechanistic hypotheses, although plausible, warrant further molecular and biochemical investigations to elucidate the specific pathways through which chromium interferes with essential ion homeostasis and photosynthetic function in aquatic macrophytes. It is also important to acknowledge that, although our focus was on Cr, other heavy metals present in tannery effluents and various organic compounds may have contributed to the observed effects. Therefore, future studies should investigate these additional contaminants to understand the environmental impacts of tannery effluents comprehensively.

Specifically, the observed reduction in levels of chlorophyll *a*, *b*, and total (Figure 2) reinforces the hypothesis that Cr directly interferes with photosynthetic pigments' biosynthesis, significantly impairing photosynthetic efficiency in *S. auriculata* . This effect has been extensively documented in the literature, where heavy metals, including Cr, have been shown to inhibit chlorophyll synthesis by interfering with the absorption and metabolism of essential nutrients such as magnesium and iron, both crucial for the formation of the porphyrin ring of the chlorophyll molecule [58, 59, 60, 61, 62]. Additionally, Cr can destabilize chloroplast membranes and interfere with the activities of enzymes involved in photosynthesis, further exacerbating chlorophyll production deficiencies and, consequently, the plant's ability to perform efficient photosynthesis [62, 63]. Notably, the maintenance of the ratio between chlorophyll *a* and *b*, despite the overall reduction in chlorophyll levels, suggests an intrinsic adaptive response of the plant. This adjustment may reflect a homeostatic strategy to maintain the functional balance between chlorophyll types, which is, as discussed by Wientjes et al. [64], essential for the efficiency of Photosystem II (PSII) and for capturing light energy under environmental stress conditions. Studies have shown that, under stress conditions induced by heavy metals, some plants can modulate the ratio of chlorophylls as a way to preserve the functional integrity of the photosynthetic apparatus, ensuring that light absorption and electron transfer occur effectively, even in adverse environments [65, 66]. Therefore, this adaptive response may be crucial for the survival of *S. auriculata* in contaminated environments, allowing the plant to maximize light capture and minimize photochemical damage, even in the presence of contaminants such as Cr.

Despite not observing significant changes in ROS and MDA levels, which are classic biomarkers of oxidative stress (Figure 3A,B), the analysis of the antioxidant enzymes SOD and CAT activities revealed substantial modulation in response to exposure to tannery effluents. The CAT/SOD ratio was significantly higher in the RTE‐3.1 group and lower in the TTE‐3.1 group (Figure 4D), suggesting a complex adjustment of the plants' antioxidant response to handle the stress imposed by the effluents. This adjustment can be understood as an attempt by the plants to modulate the balance between ROS production and elimination. As highlighted by Ighodaro and Akinloye [67], the enzyme SOD is the first line of antioxidant defense, catalyzing the dismutation of superoxide anion (O2•−) into hydrogen peroxide (H_2_O_2_). Although we did not observe differences in the isolated activity of SOD, the increase in the CAT/SOD ratio observed in the RTE‐3.1 group indicates a need for greater efficiency in hydrogen peroxide detoxification, potentially due to a trend toward increased MDA production (Figure 3B), even if not statistically significant, suggesting a slight increase in oxidative stress. However, hydrogen peroxide generated by SOD is, in itself, a potentially toxic molecule that needs to be rapidly eliminated by catalase (CAT) action, which explains the increase in the CAT/SOD ratio. The decrease in this ratio in the TTE‐3.1 group may reflect a lower need for detoxification, suggesting that exposure to treated effluent may have induced less pronounced stress or that other antioxidant pathways may have been activated.

Another interesting finding in our study refers to the significant suppression of nitrite production in plants exposed to the 3.1% diluted raw effluent (Figure 3C) and the strong negative correlation between nitrite levels and Cr accumulation in the plant fronds (Figures 7 and 9E). The reduction in nitrite levels may be directly related to Cr's interference in NO metabolism, a critical signaling molecule in plants that plays essential roles in regulating growth, development, and stress responses [68, 69]. As discussed by Crawford [70], nitrite is a key intermediate in the NO synthesis pathway, and its reduction may indicate an inhibition in NO production, possibly due to Cr toxicity. Previous studies suggest that Cr can inhibit the enzymes nitrite reductase and nitric oxide synthase, which are responsible for NO production from nitrite and other precursors, leading to a decrease in NO signaling [71, 72, 73]. Thus, it is plausible that similar mechanisms may have operated in our study, reinforcing the hypothesis that Cr may have directly interfered with the NO biosynthetic pathway, contributing to the observed suppression in nitrite levels. However, it is also important to consider that tannery effluents contain a complex mixture of other pollutants, which may also interfere with NO metabolism and, therefore, could have contributed to the observed suppression of nitrite levels.

The bioaccumulation capacity of Cr in *S. auriculata* was indirectly assessed in our study, with the results revealing that plants exposed to the 3.1% diluted effluents accumulated significantly higher concentrations of Cr in both fronds and roots (Figure 6A,B). These findings are consistent with the recent study by López Arias et al. [74], which demonstrated that *S. auriculata* has a high capacity to remove Cr(III) in hydroponic solutions and when exposed to tannery effluents, showing an average reduction of 57% in Cr(III) levels after 72 h of exposure. Similarly, Espinoza‐Quinones et al. [75] reported that after 35 days of exposure to an initial concentration of 5.0 mg/L of Cr(III), *S. auriculata* was able to remove approximately 90% of the metal. On the other hand, the higher CrF/CrR ratio in the group exposed to the 0.3% treated effluent (Figure 6C) may be attributed to the fact that the CMPs preferentially adsorbed the more readily available forms of Cr, leaving the less readily adsorbable forms in the effluent. Consequently, the remaining Cr in the effluent, although in a lower total amount, may be present in a chemical form that is more readily absorbed by the fronds than by the roots, possibly due to its greater mobility or solubility in aqueous solution. Thus, this differentiated Cr accumulation dynamic in response to exposure to effluents treated with CMPs highlights the need for further investigations to elucidate how different forms of Cr in the effluent interact with various parts of the plant and how this may influence the metal's bioavailability and toxicity.

It is important to note that although Cr accumulation was the dominant variable explaining PC1 separation (Figure 9A), its lack of direct correlation with antioxidant biomarkers such as the SOD/CAT ratio suggests that Cr‐induced stress did not primarily manifest through classical oxidative stress pathways in *S. auriculata* . Instead, Cr accumulation predominantly affected physiological traits related to growth and photosynthesis, as evidenced by strong negative correlations with chlorophyll content and “root” length. This dissociation likely reflects the plant's capacity to compartmentalize or tolerate intracellular Cr without triggering a generalized oxidative stress response, a phenomenon previously described in aquatic macrophytes exposed to heavy metals. Furthermore, antioxidant responses appeared more closely modulated by effluent concentration and chemical complexity than by Cr bioaccumulation per se, as highlighted by the distinct clustering of antioxidant biomarkers in the multivariate analyses.

The integration of the PCA results (Figure 9A) and the Cluster analysis (Figure 9B) reinforces and synthesizes the complex interactions between tannery effluents and the responses of *S. auriculata* observed throughout our study. The PCA highlighted a clear separation between the groups exposed to raw and treated effluents, suggesting that Cr bioaccumulation and its physiological consequences, such as reduced photosynthesis and altered antioxidant response, are key factors in distinguishing between the groups. This separation indicates that Cr, even after effluent treatment, continues to play a central role in modulating the plant's biochemical responses, reinforcing the idea that treatment with CMPs, while effective in reducing the total Cr concentration, does not completely eliminate its toxic effects. The Cluster analysis, in turn, complements these findings by revealing coherent groupings of physiological responses according to different treatments, suggesting that the groups exposed to the 0.3% treated effluent exhibit a distinct response, possibly due to the chemical form of the residual Cr after adsorption by the CMPs. These grouping patterns reflect the complexity of interactions between the various contaminants present in tannery effluents and their different bioavailabilities and toxicities. Thus, the multivariate analyses not only corroborate previous findings but also emphasize the need for future investigations that address the interactions between multiple contaminants in effluents and their implications for ecotoxicity in aquatic ecosystems. Such studies will be crucial for developing more effective approaches to effluent treatment and mitigating their environmental impacts.

Ultimately, from a practical perspective, the findings of this study offer valuable insights into industrial effluent treatment strategies and environmental regulations. Although the application of MCPs led to partial reductions in Cr concentrations and mitigated certain toxicological endpoints in *S. auriculata* , the treatment was not fully effective. This partial efficacy could be attributed to factors such as insufficient contact time, saturation of adsorption sites, or the presence of other co‐contaminants that interact with the cellulose matrix. Optimizing operational parameters, such as increasing the surface area of the MCPs, enhancing the functionalization of the material, or combining it with other bio‐based adsorbents, may significantly improve removal efficiency. These considerations highlight the importance of developing adaptable, low‐cost, and scalable technologies for tannery effluent remediation. Moreover, the use of aquatic macrophytes as sentinels for ecotoxicological assessment could assist regulatory agencies in monitoring treated effluents and supporting policy decisions that align industrial practices with environmental sustainability goals.

## 5 Limitations and Future Perspectives

Although our study has provided valuable insights into the ecotoxicological effects of tannery effluents on *S. auriculata* , several limitations should be considered when interpreting the results. One of the main limitations is the inherent complexity of tannery effluents, which contain a mixture of heavy metals, such as chromium, and a variety of organic compounds whose presence and concentration were not fully characterized in our study. This complexity hinders a more detailed assessment of the interactions between the different pollutants and their combined effects on the plants. In this context, it is important to consider that the toxicological effects observed may result not only from the action of individual contaminants but also from synergistic interactions between multiple substances, which could enhance toxicity in ways that are difficult to predict using isolated approaches. Therefore, future studies should incorporate chemical profiling and mixture toxicity evaluations to better reflect environmentally relevant scenarios. Additionally, while we focused on Cr bioaccumulation, the possible presence of other metals, such as Zn and Cu, as well as organic compounds, may have influenced the observed physiological responses, underscoring the need for future studies to incorporate a more comprehensive analysis of the effluent composition and its effects.

Another aspect to consider is the use of *S. auriculata* as a model organism. Although this species is an excellent bioindicator due to its ability to accumulate heavy metals, the results obtained in our study may not be directly extrapolated to other aquatic organisms or field conditions. The physiological responses of different species can vary significantly, depending on their biological and ecological characteristics, as well as the environmental conditions to which they are subjected. Consequently, the observed effects on *S. auriculata* could potentially influence broader ecosystem processes, such as primary productivity, nutrient cycling, or habitat structure, particularly in environments where this macrophyte plays a key ecological role. Thus, supplementary studies using a broader range of aquatic organisms and considering environmental variables, such as temperature, pH, and nutrient concentration, would be essential to validate and expand upon the findings of our study.

It will also be important to assess the effects of effluents at other dilutions beyond those tested in our study. While the dilutions we evaluated are considered predictive and environmentally relevant, investigating ecotoxicity across a broader range of concentrations could provide a more comprehensive view of the risks associated with different levels of exposure. Moreover, the exposure duration used in our study, 15 days, may not have been sufficient to capture chronic or sublethal effects that could manifest with prolonged exposure. Finally, while our study included a detailed analysis of biomarkers such as chlorophyll, ROS, MDA, and the antioxidant enzymes SOD and CAT, incorporating additional biomarkers could enhance the understanding of plant toxicity and adaptation mechanisms. Biomarkers related to DNA damage (e.g., the comet assay), gene expression of antioxidant defense proteins (e.g., heat shock proteins, HSPs), and key enzymes involved in stress responses could provide further insights into the adaptive mechanisms proposed in our study. Additionally, measuring physiological biomarkers such as transpiration rate, stomatal conductance, leaf water potential, and enzymes related to the Calvin cycle (e.g., ribulose‐1,5‐bisphosphate carboxylase/oxygenase, RuBisCO) could also elucidate the processes of plant adaptation in response to stress caused by effluents. The inclusion of these biomarkers would not only complement our findings and reinforce the hypotheses raised but also help clarify the involved toxicity pathways, allowing the identification of critical intervention points to mitigate the adverse effects of effluents.

## 6 Conclusion

Based on our results, we conclude that although the treatment of tannery effluents with CMPs led to partial reductions in toxicity, the treated effluents still caused significant physiological and biochemical alterations in *S. auriculata* . These effects included inhibition of root growth, pigment degradation, oxidative imbalance, and chromium bioaccumulation, indicating that the treatment was not fully effective in mitigating the adverse impacts of the effluent. Therefore, our findings challenge the initial hypothesis that treated effluents would have substantially lower toxicity, highlighting the need to incorporate ecotoxicological criteria when evaluating the performance of effluent treatment technologies. While CMPs show promise as an environmentally friendly and low‐cost adsorbent, their optimization is necessary to improve the removal efficiency of chromium and other co‐occurring pollutants. These findings contribute to the understanding of treatment limitations and provide necessary guidance for industries and regulatory bodies seeking to develop safer and more sustainable effluent management practices. Further research is essential to assess chronic effects, evaluate a broader range of contaminants and organisms, and refine treatment strategies for real‐world applications.

## Author Contributions

**Alex Rodrigues Gomes:** conceptualization, methodology, investigation, supervision. **Letícia Paiva de Matos:** conceptualization, methodology, investigation, supervision, writing – original draft. **Abner Marcelino Silva:** methodology, investigation. **Abraão Tiago Batista Guimrães:** investigation. **Thiarlen Marinho da Luz:** investigation. **Rafaela Ribeiro de Brito:** investigation. **Aline Sueli Lima de Rodrigues:** review. **Ivandilson Pessoa Pinto de Menezes:** review. **Juraci Alves de Oliveira:** review. **Guilherme Malafaia:** contextualization, methodology, validation, formal analysis, resources, data curation, writing – original draft, review and editing, supervision, project administration, funding acquisition.

## Ethics Statement

The authors have nothing to report.

## Consent

The authors have nothing to report.

## Conflicts of Interest

We confirm that there are no conflicts of interest or financial support that could have influenced this work. All authors have read and approved the manuscript, agree on the authorship order, and no eligible contributors have been omitted. The integrity of the work was ensured throughout. The reviewers assigned to this manuscript were selected solely by the handling Editor, with no involvement or responsibility from the corresponding author.

## Data Availability

The data that support the findings of this study are available from the corresponding author upon reasonable request.
